# Supplementary material for: Robust data storage in DNA by de Bruijn graph-based de novo strand assembly
Source: Nat Commun. 2022 Sep 12;13:5361. doi: 10.1038/s41467-022-33046-w (PMC9468002; doi:10.1038/s41467-022-33046-w)
Supplement: Supplementary file 2 — Description of Additional Supplementary Files [file 41467_2022_33046_MOESM2_ESM.pdf]

Title: Supplementary Movie 1
Description: End-to-end presentation of the decoding process of DBGPS and outer fountain codes.
The decoding started with raw sequencing reads encoding a 6.8 MB zipped file of ten pictures of Dunhuang murals. The decoding includes two major steps. At first, the raw sequencing reads are handled by DBGPS, generating strand sequences. Then, the DNA fountain codes are then utilized to decipher the original data. At the end of the movie, the decoded file was checked by comparing the MD5 hash values and opening the unzipped pictures manually.

Title: Supplementary Data 1
Description: The 6.8 MB zipped file used as input data in the robustness verification experiments.

Title: Supplementary Data 2
Description: The design sequences of the 21,000 DNA strands encoding the 6.8 MB zipped file.
