# Supplementary figures and images for: Robust data storage in DNA by de Bruijn graph-based de novo strand assembly
Source: Nat Commun. 2022 Sep 12;13:5361. doi: 10.1038/s41467-022-33046-w (PMC9468002; doi:10.1038/s41467-022-33046-w)

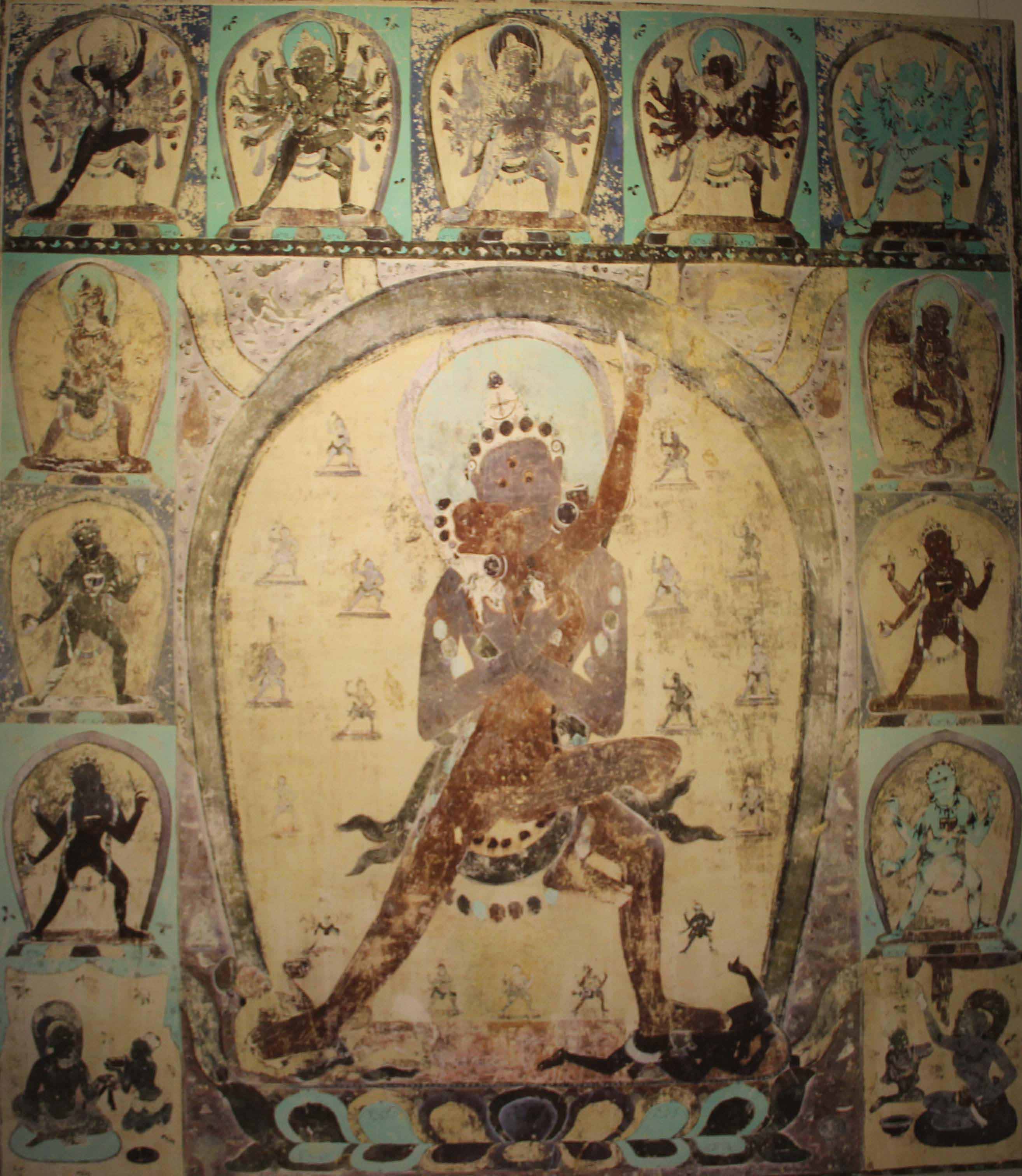

Supplement: Supplementary file 4 — Supplementary Data 1 [file 41467_2022_33046_MOESM4_ESM.zip › 01.jpg]

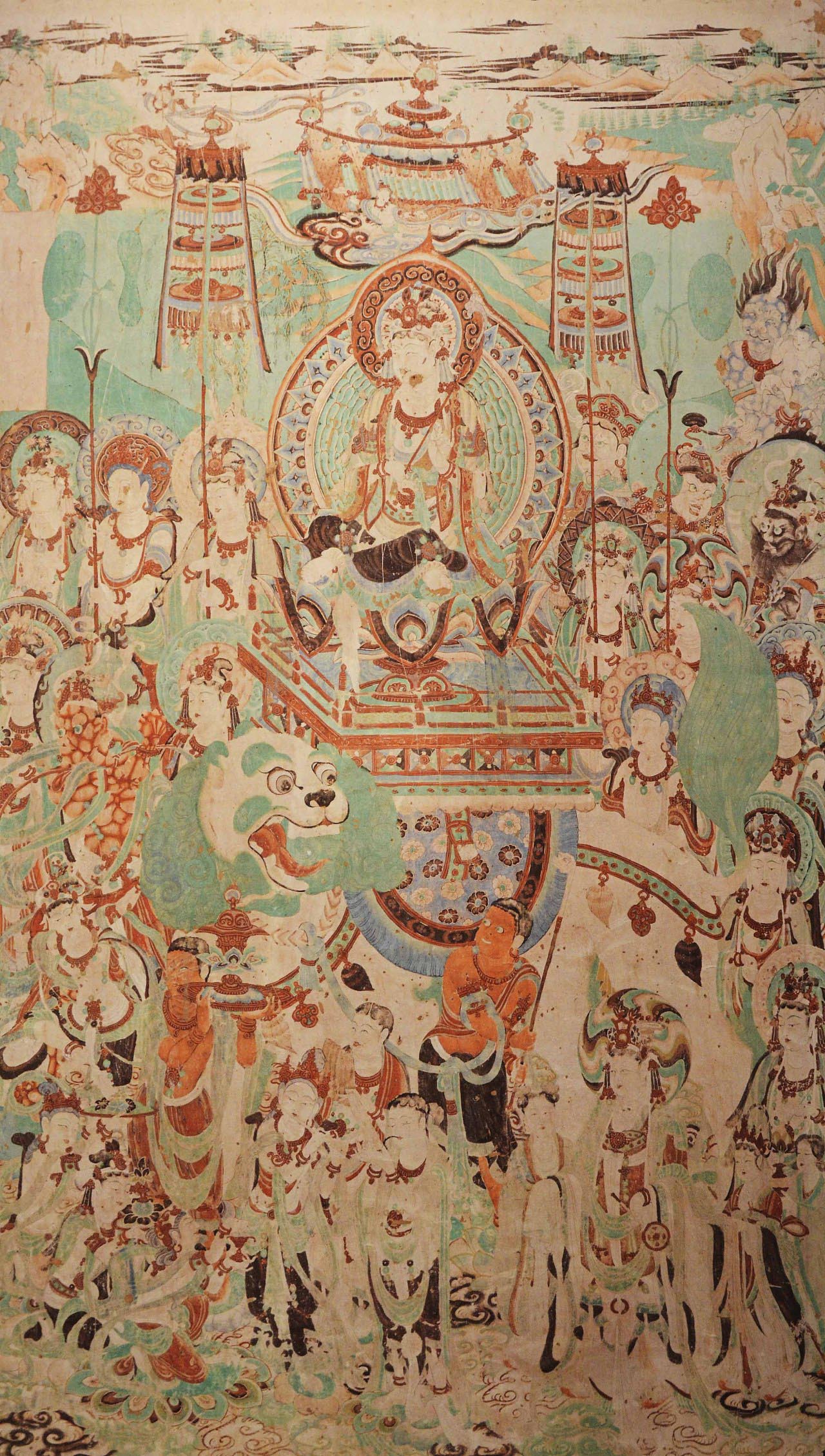

Supplement: Supplementary file 4 — Supplementary Data 1 [file 41467_2022_33046_MOESM4_ESM.zip › 02.jpg]

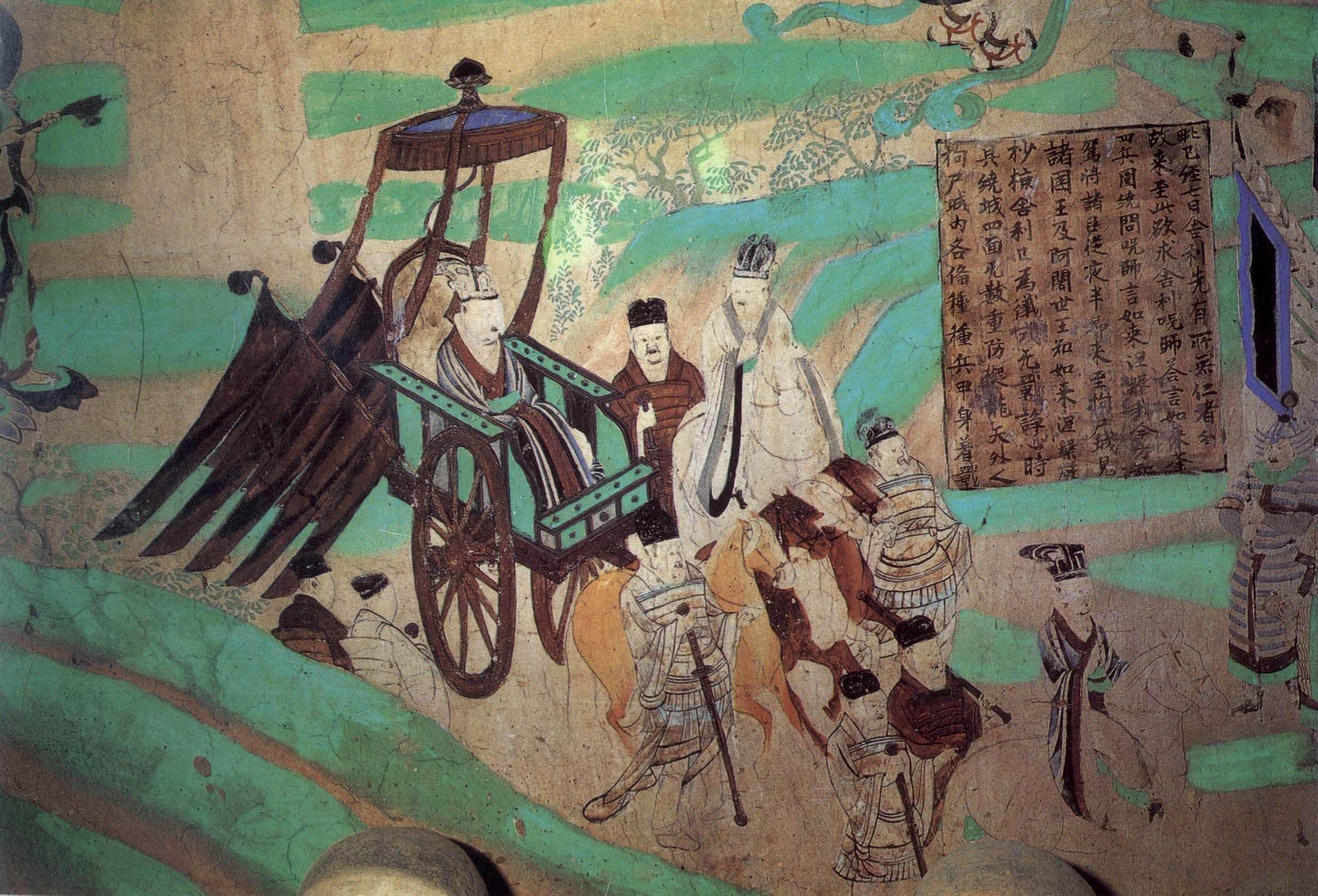

Supplement: Supplementary file 4 — Supplementary Data 1 [file 41467_2022_33046_MOESM4_ESM.zip › 03.jpg]

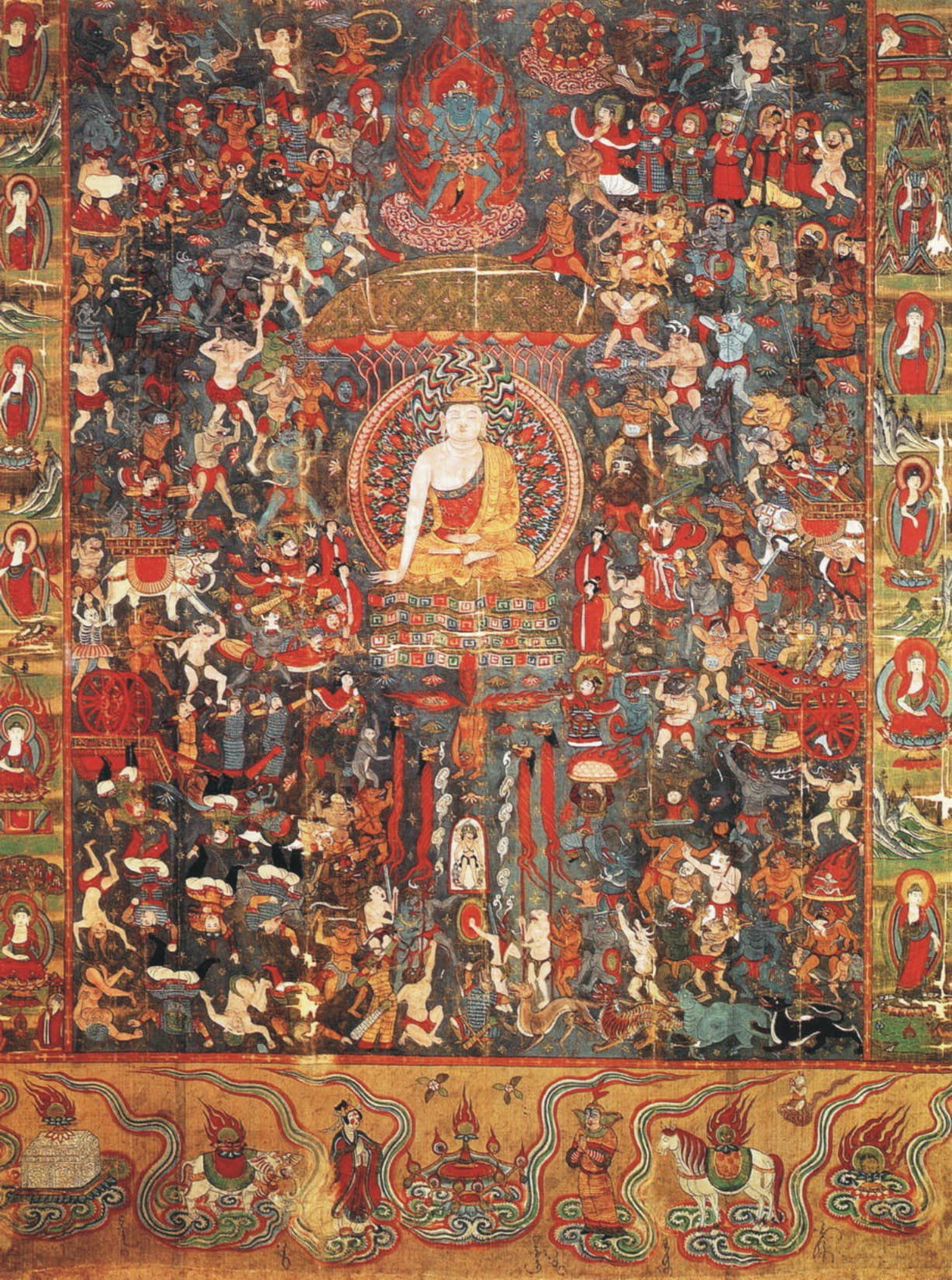

Supplement: Supplementary file 4 — Supplementary Data 1 [file 41467_2022_33046_MOESM4_ESM.zip › 04.jpg]

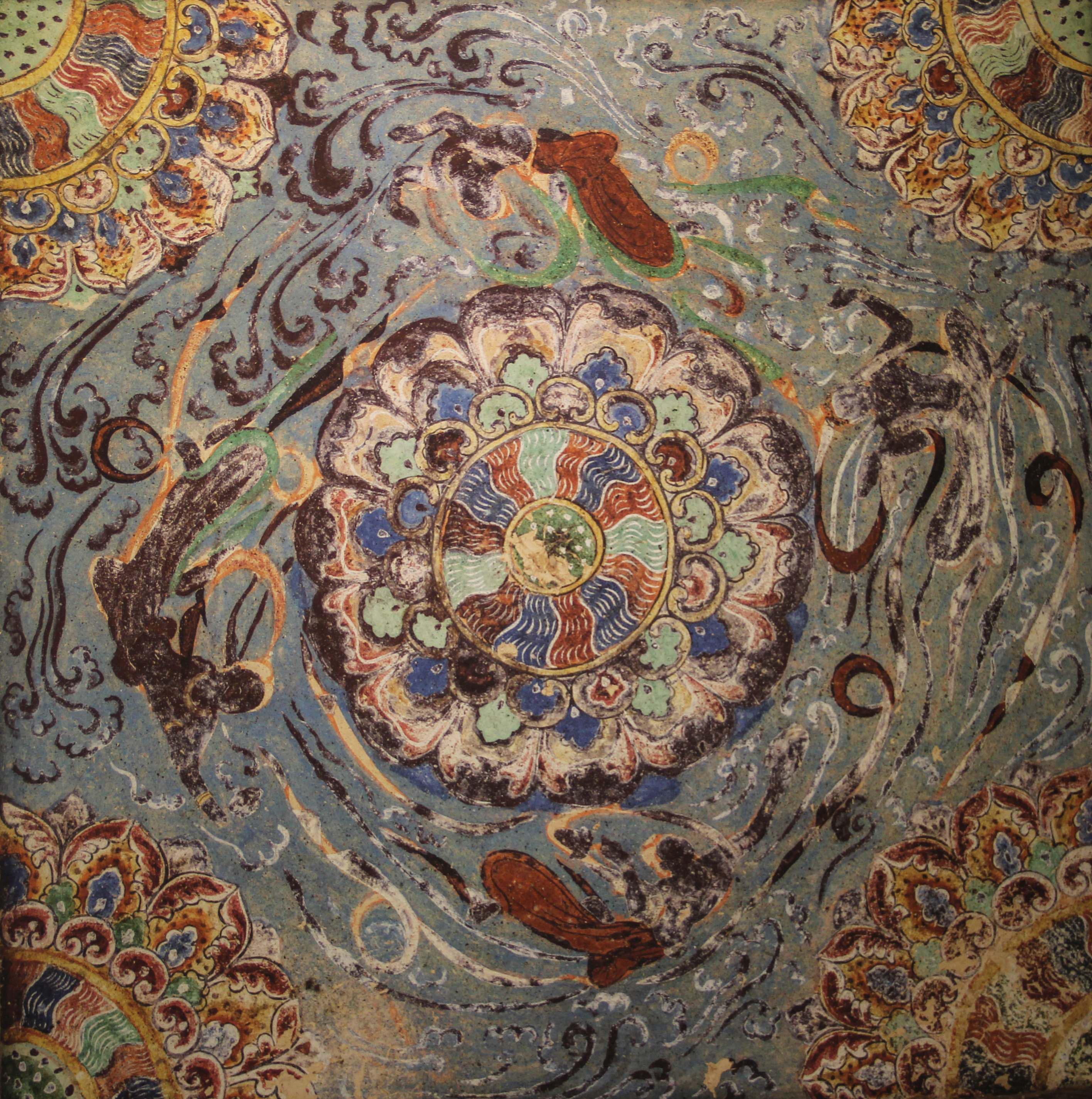

Supplement: Supplementary file 4 — Supplementary Data 1 [file 41467_2022_33046_MOESM4_ESM.zip › 05.jpg]

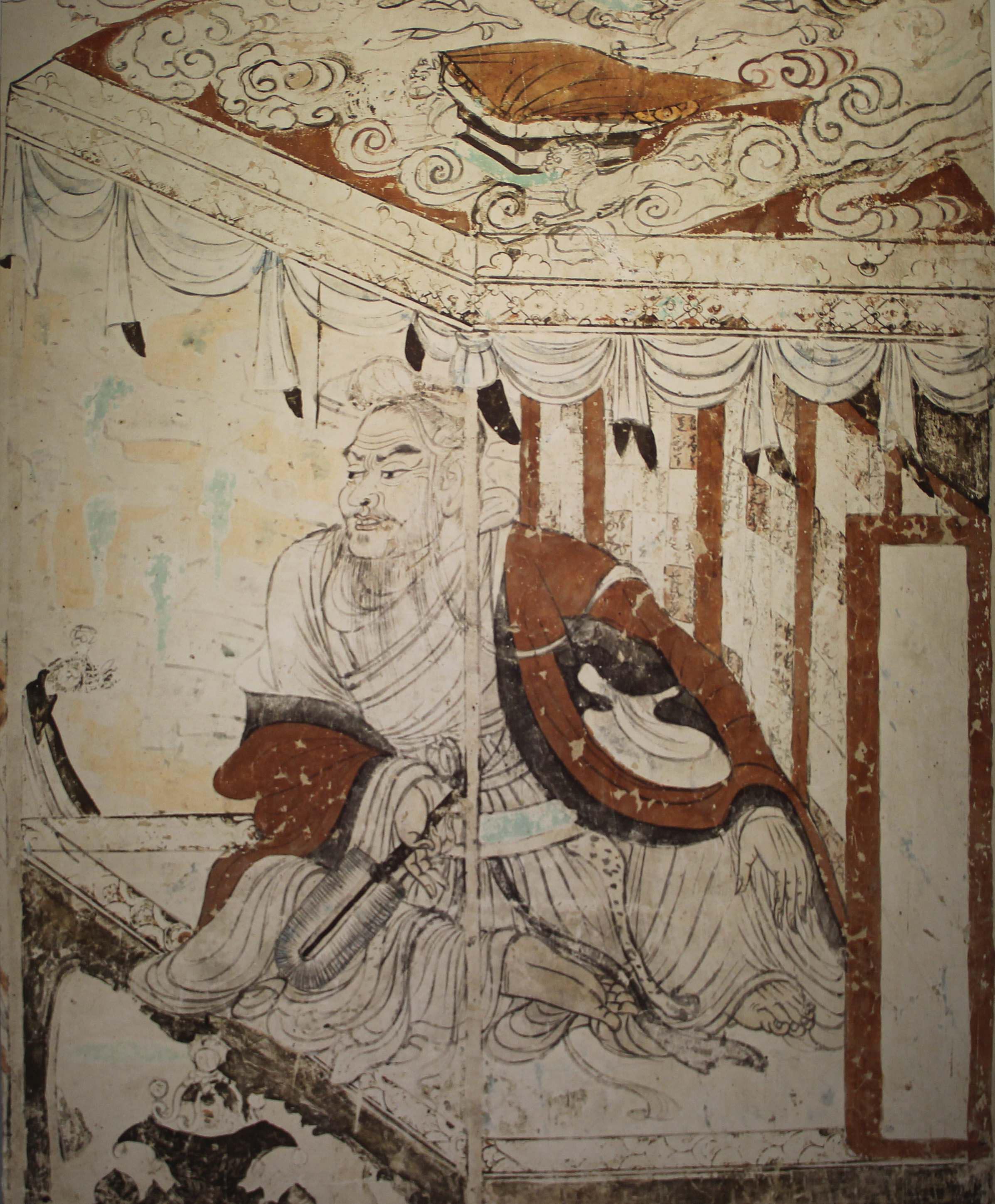

Supplement: Supplementary file 4 — Supplementary Data 1 [file 41467_2022_33046_MOESM4_ESM.zip › 06.jpg]

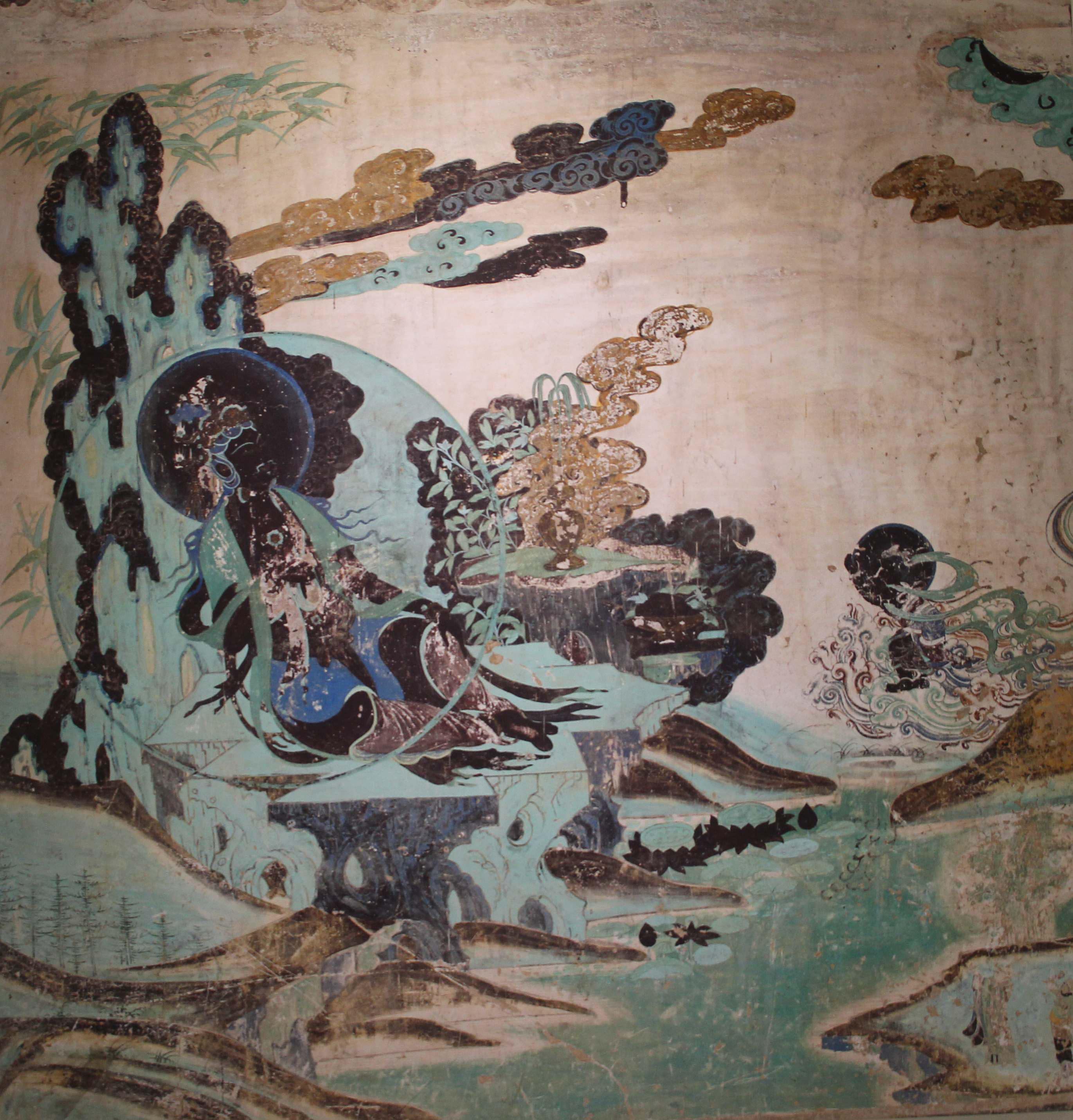

Supplement: Supplementary file 4 — Supplementary Data 1 [file 41467_2022_33046_MOESM4_ESM.zip › 07.jpg]

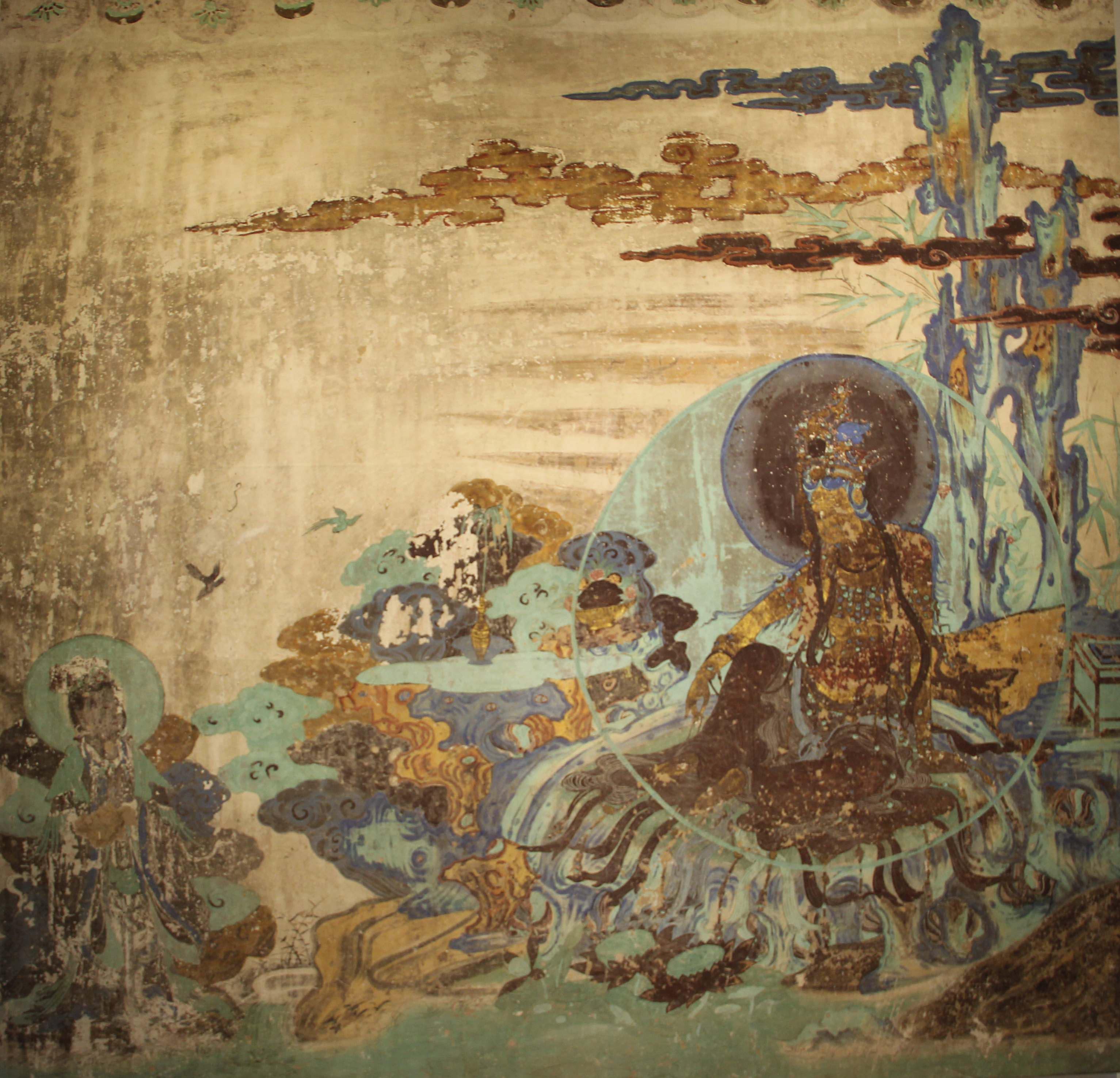

Supplement: Supplementary file 4 — Supplementary Data 1 [file 41467_2022_33046_MOESM4_ESM.zip › 08.jpg]

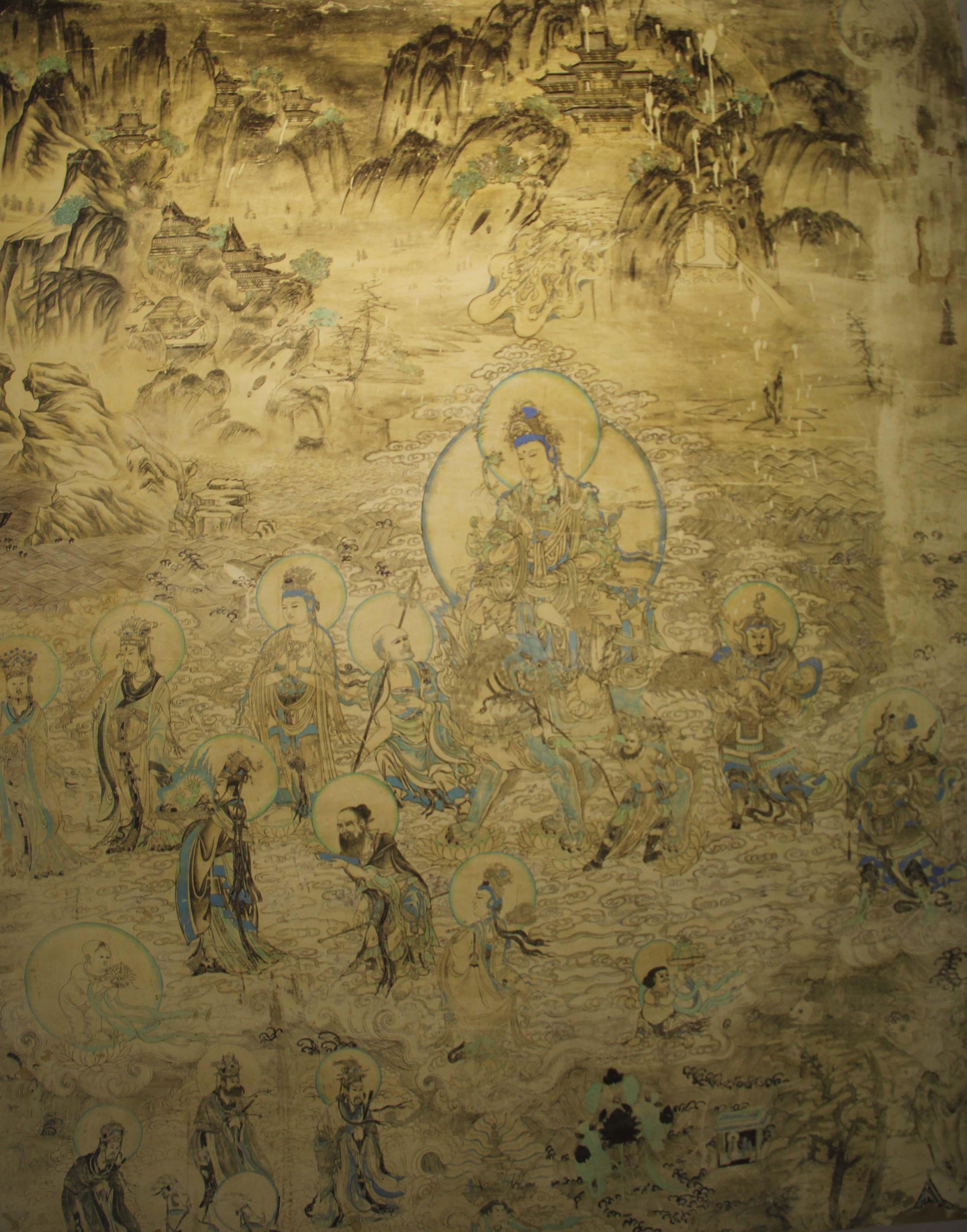

Supplement: Supplementary file 4 — Supplementary Data 1 [file 41467_2022_33046_MOESM4_ESM.zip › 09.jpg]

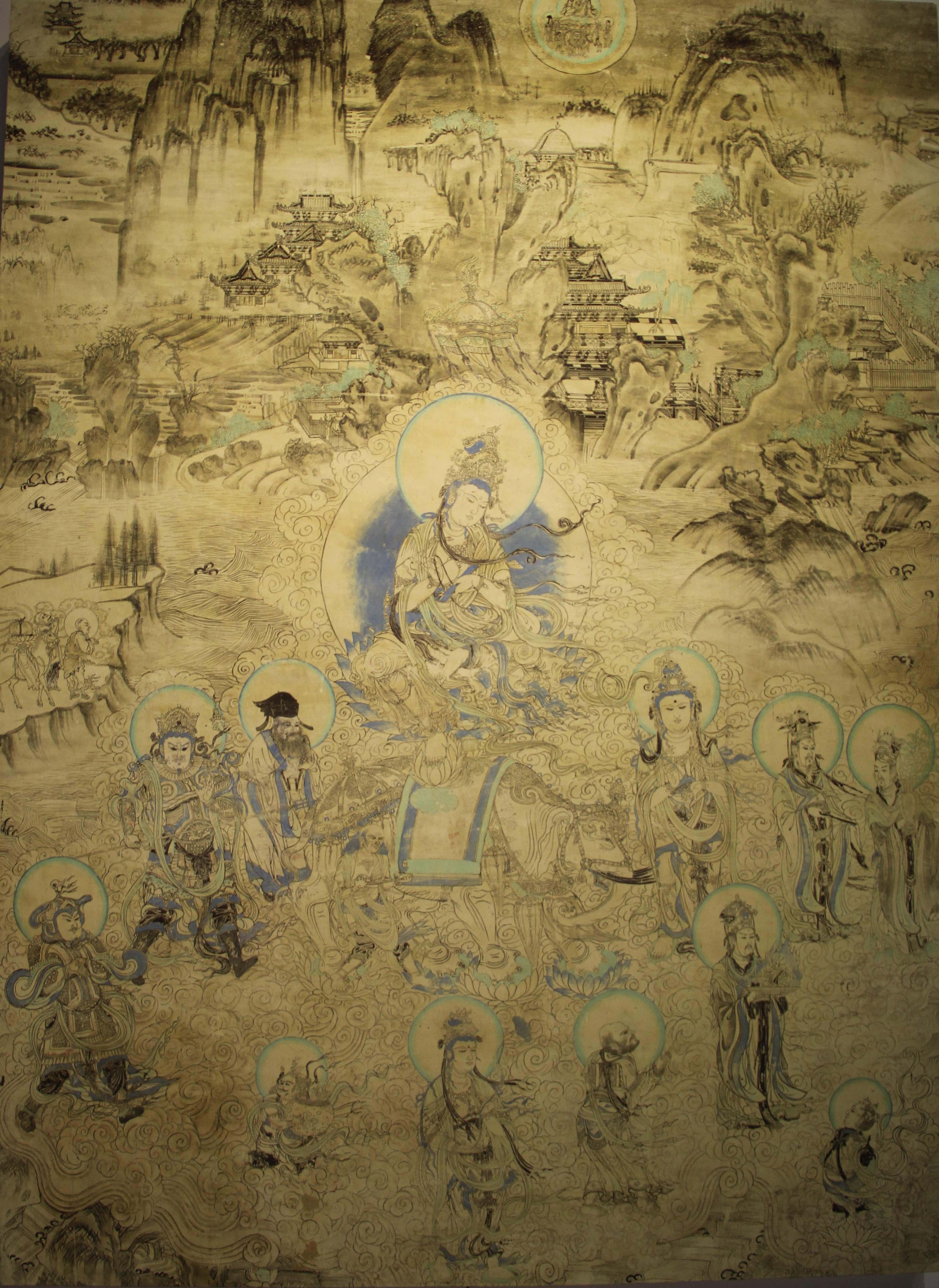

Supplement: Supplementary file 4 — Supplementary Data 1 [file 41467_2022_33046_MOESM4_ESM.zip › 10.jpg]
