# Supplementary material for: Robust data storage in DNA by de Bruijn graph-based de novo strand assembly
Source: Nat Commun. 2022 Sep 12;13:5361. doi: 10.1038/s41467-022-33046-w (PMC9468002; doi:10.1038/s41467-022-33046-w)
Supplement: Supplementary file 7 — Reporting Summary [file 41467_2022_33046_MOESM7_ESM.pdf]

nature research

Corresponding author(s): Bingzhi Li, Yingjin Yuan

Last updated by author(s): Aug 2, 2022

Reporting Summary

Nature Research wishes to improve the reproducibility of the work that we publish. This form provides structure for consistency and transparency in reporting. For further information on Nature Research policies, see our Editorial Policies and the Editorial Policy Checklist.

Statistics

For all statistical analyses, confirm that the following items are present in the figure legend, table legend, main text, or Methods section.

n/a	Confirmed	
☐	☒	The exact sample size (n) for each experimental group/condition, given as a discrete number and unit of measurement
☐	☒	A statement on whether measurements were taken from distinct samples or whether the same sample was measured repeatedly
☒	☐	The statistical test(s) used AND whether they are one- or two-sided<br>*Only common tests should be described solely by name; describe more complex techniques in the Methods section.*
☐	☒	A description of all covariates tested
☐	☒	A description of any assumptions or corrections, such as tests of normality and adjustment for multiple comparisons
☐	☒	A full description of the statistical parameters including central tendency (e.g. means) or other basic estimates (e.g. regression coefficient) AND variation (e.g. standard deviation) or associated estimates of uncertainty (e.g. confidence intervals)
☒	☐	For null hypothesis testing, the test statistic (e.g. F, t, r) with confidence intervals, effect sizes, degrees of freedom and P value noted<br>*Give P values as exact values whenever suitable.*
☐	☒	For Bayesian analysis, information on the choice of priors and Markov chain Monte Carlo settings
☐	☒	For hierarchical and complex designs, identification of the appropriate level for tests and full reporting of outcomes
☒	☐	Estimates of effect sizes (e.g. Cohen's d, Pearson's r), indicating how they were calculated

Our web collection on statistics for biologists contains articles on many of the points above.

Software and code

Policy information about availability of computer code

Data collection	-The outer codes is modified from https://github.com/dbieber/fountaincode<br>-The ten Dunhuang mural images were obtained from Dunhuang Academy (http://www.dha.ac.cn/) with permissions usage in this publication.
Data analysis	-The python codes implemented in this study is available under: https://doi.org/10.5281/zenodo.6833784<br>-A compiled C implementation of DBGPS is available at https://doi.org/10.5281/zenodo.6833747<br>-For DNA sequence multiple alignments we used Muscle 3.8.31 and mafft v7.490.<br>-For paired-end read assembly, we used Pear v0.9.11 and Flash v1.2.11.<br>-For clustering of reads, we used Starcode v1.0.<br>-For NGS data analysis of FastQ files, we used FastP v0.23.2.<br>-BLAST+ 2.2.27 was used in the sequencing data analysis.<br>-Jellyfish 2.3 was used for the counting of k-mer sin large-scale simulations.<br>-pigz v1.2.3 was used to generate the zip file for large scale simulations.

For manuscripts utilizing custom algorithms or software that are central to the research but not yet described in published literature, software must be made available to editors and reviewers. We strongly encourage code deposition in a community repository (e.g. GitHub). See the Nature Research guidelines for submitting code & software for further information.

Data

Policy information about availability of data

All manuscripts must include a data availability statement. This statement should provide the following information, where applicable:

- Accession codes, unique identifiers, or web links for publicly available datasets
- A list of figures that have associated raw data
- A description of any restrictions on data availability

Source data are provided with this paper.
The sequencing data generated in this study have been deposited in the figshare database under the following DOI links:
Accelerated aging samples of 70¬°C for 0 and 28 days. https://doi.org/10.6084/m9.figshare.17193170.v2
Accelerated aging samples of 70¬°C for 56 and 70 days. https://doi.org/10.6084/m9.figshare.17192639.v1
Three samples of the 100 independent retrievals. https://doi.org/10.6084/m9.figshare.18515078.v1
Error-prone PCR 1st and 2st rounds. https://doi.org/10.6084/m9.figshare.16727122.v2
Error-prone PCR 3st and 4st rounds. https://doi.org/10.6084/m9.figshare.17193128.v1
Error-prone PCR 5st and 6st rounds. https://doi.org/10.6084/m9.figshare.18515045.v1
High density storage - 295PB/g. https://doi.org/10.6084/m9.figshare.17183081.v1
The uniref90 database was downloaded at https://ftp.uniprot.org/pub/databases/uniprot/uniref/uniref90/uniref90.fasta.gz

Field-specific reporting

Please select the one below that is the best fit for your research. If you are not sure, read the appropriate sections before making your selection.

☒ Life sciences ☐ Behavioural & social sciences ☐ Ecological, evolutionary & environmental sciences

For a reference copy of the document with all sections, see nature.com/documents/nr-reporting-summary-flat.pdf

Life sciences study design

All studies must disclose on these points even when the disclosure is negative.

Sample size	For data retrieval, we always send the whole sample for sequencing. The coverages of the sequencing reads were determined by the input data size and error rates of the DNA sample. A coverage >10 is normally required. The accurate data retrievals confirms the proper read sizes are chosen.
Data exclusions	Raw sequencing data were directly applied in all decoding experiments.
Replication	The decoding process were repeated more than 100 times. The encoded data was decoded correctly in all repeats.
Randomization	Random errors are introduced experimentally and by simulation.
Blinding	The DBGPS decoder rebuilds the strand sequences in a blind way.

Reporting for specific materials, systems and methods

We require information from authors about some types of materials, experimental systems and methods used in many studies. Here, indicate whether each material, system or method listed is relevant to your study. If you are not sure if a list item applies to your research, read the appropriate section before selecting a response.

Materials & experimental systems

n/a	Involved in the study	
☒	☐	Antibodies
☒	☐	Eukaryotic cell lines
☒	☐	Palaeontology and archaeology
☒	☐	Animals and other organisms
☒	☐	Human research participants
☒	☐	Clinical data
☒	☐	Dual use research of concern

Methods

n/a	Involved in the study	
☒	☐	ChIP-seq
☒	☐	Flow cytometry
☒	☐	MRI-based neuroimaging
